# In Memoriam—Henry Noah Martin, M. D.

**Published:** 1889-12

**Authors:** 


### IN MEMORIAM—HENRY NOAH MARTIN, M. D.,

Preamble and Resolutions by the Bœnninghausen Medical Club, of Philadelphia.

Death having severed the pleasant relationship that existed between Henry Noah Martin, M. D., and ourselves, we respectfully resolve—

1st. That during his life he was an earnest worker, a safe adviser, and a valued friend, and by his death Homœopathy has lost one of its most strenuous advocates and honest believers.

2d. That we extend to his family and to the community our sincere sympathy for the loss of one who was so genial, kind, and brilliant.

3d. That a copy of these resolutions be sent to his wife; that they be printed in the *Hahnemannian Monthly* and HOMŒOPATHIC PHYSICIAN of Philadelphia; and that they be entered in the Journal of this Society.

GEO. S. PARKE, M. D.,

GEO. W. SMITH, M. D.,

*Committee.*

